# The effect of oral nutritional supplement therapy on nutritional status and quality of life in patients with esophageal cancer undergoing radiotherapy and chemotherapy: A protocol for randomized controlled trial

**DOI:** 10.1097/MD.0000000000025342

**Published:** 2021-04-23

**Authors:** Yunxia Chen, Xiancui Wu, Xiaowei Wei, Li Xu, Xiuqin Ren

**Affiliations:** Department of Oncology, Nanjing First Hospital, Nanjing Medical University, Nanjing, Jiangsu Province, China.

**Keywords:** esophageal cancer, oral nutritional supplement therapy, protocol, radiotherapy and chemotherapy, randomized controlled trial

## Abstract

**Background:**

The incidence of malnutrition in patients with esophageal cancer is high, which seriously affects the therapeutic effect and quality of life. Oral nutritional supplement is the first choice of nutritional support recommended by current guidelines, which can supplement the lack of energy and protein in patients with esophageal cancer, improve nutritional status and improve the quality of life, but there are few clinical studies. Therefore, the purpose of this randomized controlled trial is to evaluate the effect of oral nutritional supplement therapy on nutritional status and quality of life in patients with esophageal cancer treated undergoing radiotherapy and chemotherapy.

**Methods:**

This is a prospective randomized controlled trial to study the effects of oral nutritional supplement therapy on nutritional status and quality of life in patients with esophageal cancer undergoing radiotherapy and chemotherapy. This study is approved by the Clinical Research Society of our hospital. Patients will be randomly divided into ONS group and traditional diet group. The nutritional status, quality of life score and adverse reactions will be observed before and after radiotherapy and chemotherapy. The data will be analyzed by SPSS 16.0.

**Discussion:**

This study will evaluate the effect of oral nutritional supplement therapy on nutritional status and quality of life of patients with esophageal cancer undergoing radiotherapy and chemotherapy. The results of this experiment will establish clinical evidence for the application of oral nutritional supplement therapy in patients with esophageal cancer undergoing radiotherapy and chemotherapy.

**OSF Registration number:**

DOI 10.17605/OSF.IO/9ZW34.

## 1 Introduction

About 40% to 80% of patients with malignant tumors have nutrition-related problems, which are more common in patients with head and neck tumors and digestive tract tumors,^[1]^ and the patients with esophageal cancer have a higher incidence of malnutrition. It is reported that 60% to 85% of patients with esophageal cancer have different degrees of malnutrition, ranking first among all tumors.^[2]^ Malnutrition will affect the therapeutic effect of malignant tumor patients, reduce the quality of life, shorten the survival time, and increase medical expenses.^[3,4]^ According to the guidelines on enteral nutrition for tumor patients issued by the European Society of Enteral and Parenteral Nutrition in 2006, oral nutrition supplement (ONS) is recommended as the first choice of nutritional support for radiotherapy patients.^[5]^ ONS is also recommended as the first choice of enteral nutrition in the consensus of Chinese experts on enteral nutrition for radiotherapy patients with esophageal cancer undergoing radiotherapy.^[6]^

The 2006 European Society for Clinical Nutrition and Metabolism (ESPEN) guidelines unify the English full name of ONS as “oral nutritional supplements” and define it as "supplementary oral intake of foods for special medical uses (formulations) in addition to normal foods.^[7]^ Formula food for special medical purposes is specially processed to meet the special needs of nutrients or diet of people with limited intake, digestive and absorption disorders, metabolic disorders or specific disease status.^[8]^ ONS can supplement the energy and protein in patients with esophageal cancer and improve their nutritional status. At present, this conclusion has been confirmed, but there is a lack of strict randomized controlled trials (RCTs). At the same time, no study has focused on the changes in patients’ quality of life before and after treatment. This study will evaluate the effect of ONS on nutritional status and quality of life in patients with esophageal cancer treated undergoing radiotherapy and chemotherapy.

## 2 Materials and methods

### 2.1 Study design

**Figure 1:**
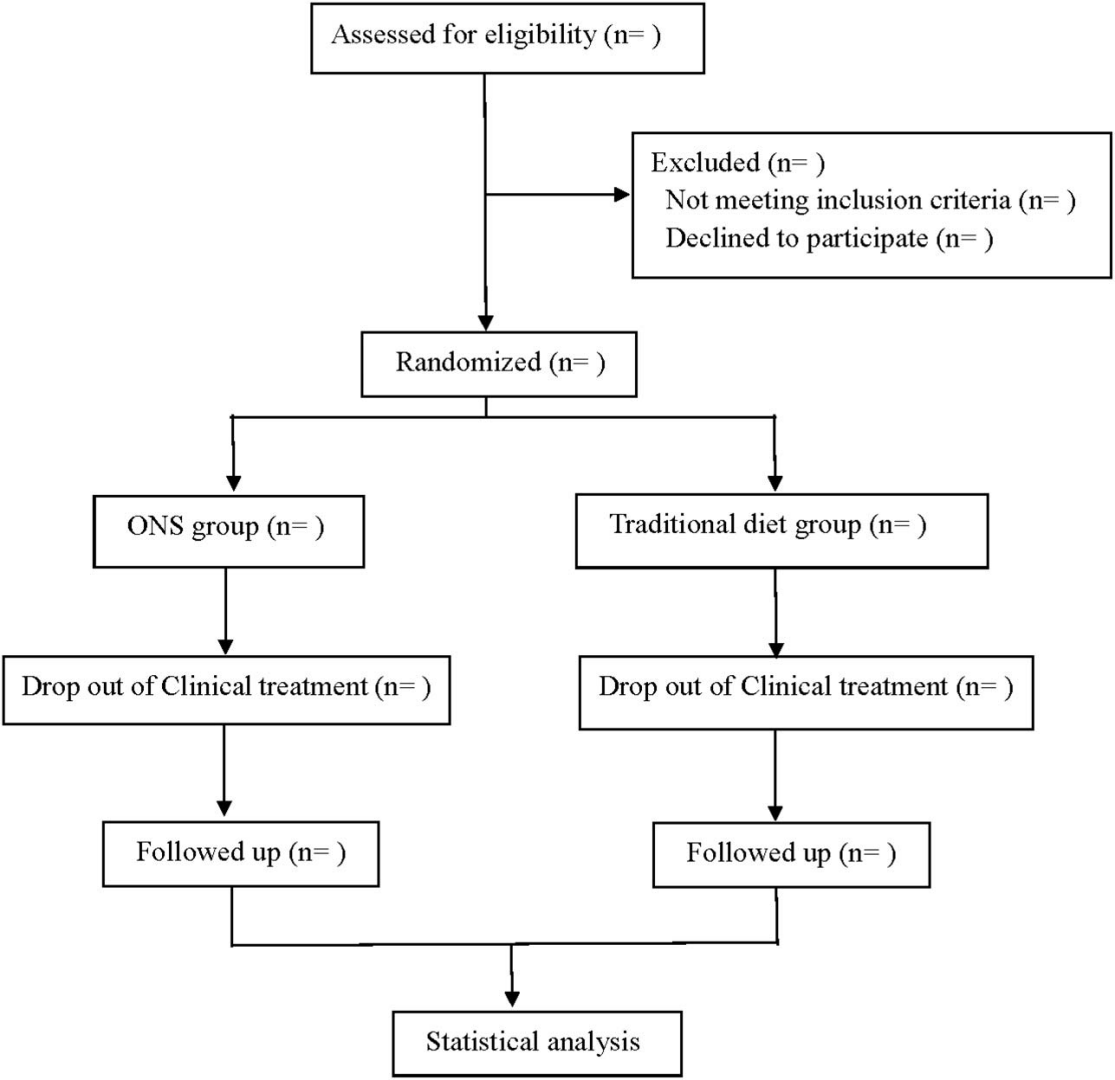
Flow diagram.

This is a prospective RCT to study the effect of oral nutritional supplement therapy on nutritional status and quality of life in patients with esophageal cancer undergoing radiotherapy and chemotherapy. We followed the Consolidated Standards of Reporting Trials (CONSORT) guidelines for reporting randomized trials and provided a CONSORT flow diagram (Fig. 1).

### 2.2 Ethics and registration

This protocol is in line with the Helsinki Declaration and approved by the Clinical Research Ethics Committee of our hospital. This experiment has been registered in the open science framework (OSF) (registration number: DOI 10.17605/OSF.IO/9ZW34). Before randomization, all patients will sign a written informed consent. and they can freely choose whether to continue the trial at any time.

### 2.3 Patients

Inclusion criteria:

(1) patients were diagnosed as esophageal cancer, without contraindications of radiotherapy and chemotherapy, planned for radiotherapy and chemotherapy;
(2) conscious, able to eat by mouth, volunteer to participate in this clinical study;
(3) Nutritional Risk Screening score ≥ 3 (NRS 2002).

Exclusion criteria:

1) patients with unstable vital signs;
2) patients with severe stress state, intractable vomiting, and severe diarrhea;
3) patients with severe malabsorption syndrome;
4) patients with mental or consciousness disorders that could not communicate;
5) unable to understand the study plan or unwilling participants after explanation.

### 2.4 Randomization and blinding

This study will be randomized into groups without any stratification. Through simple randomization, the patients in the study will be divided into the observation group and the control group according to the 1:1 ratio. Due to the limitation of the intervention in this study, this study cannot achieve strict double-blindness. We will ensure that the study designer, allocators, data analysts, intervention supervisors, and some researchers are blind to personnel allocation.

### 2.5 Sample size calculation

This study is a pilot clinical trial. The calculation of the sample size is based on the withdrawal rate of 20% in the clinical study, taking *α* *=* *0.025, power* *=* *90%,* referring to the previous literature.^[9]^ According to the albumin level after radiotherapy, the average score of the test group is 33.3, and the standard deviation is 2.68. The average of the control group is 28.5, and the standard deviation is 3.83. It will be calculated that the minimum number of patients would be included in each group is 25.

### 2.6 Intervention measures

After the beginning of the study, dietitians will give one-to-one nutrition education to the two groups of patients once a week, investigate the results of patients’ dietary intake, give nutritional guidance and formulate dietary recipes according to different conditions, and issue a diet guidance sheet for cancer patients at the same time.

In this study, patients in the control group will not be given ONS supplement. We will set up the Nutrition Support Steam to provide comprehensive nutritional care for patients in the experimental group,^[10]^ refer to the recommendation of enteral nutrition guidelines made by ESPEN,^[11]^ and make rational use of ONS for a nutritional supplement to achieve the purpose of nutritional treatment. According to the clinical nutrition diagnosis and treatment process, the nutrition support group carries on the screening, evaluation, nutrition support plan formulation, implementation, monitoring, and program adjustment. The target nutritional feeding amount was determined as follows: total energy supply was 125 to 146 KJ/ (Kg·d) and protein 1.2 to 1.5 g/ (Kg·d). Nutrition is based on the 5-step treatment principle of malnutrition,^[12]^ the individualized diet plan is made according to the different conditions of patients every day and various laboratory indicators, and the tumor total nutrition formula food is used for ONS to achieve the target requirement. The implementation of the program will begin 3 days before radiotherapy and chemotherapy.

### 2.7 Outcome measures

The related indexes of the 2 groups will be detected before and after radiotherapy and chemotherapy, and the relevant data will be recorded.

Energy intake: the dietitian uses the 24 hours retrospective method to conduct a dietary survey once a week, including the type and quantity of diet. The daily energy intake is calculated according to the Chinese Food composition Table 2002, and the body mass is measured once a week.

Hematological determination: the levels of leukocyte, hemoglobin, platelet, total protein, albumin, prealbumin, and transferrin were detected after fasting venous blood collection.

Quality of life assessment: SF-36 score (the MOS item short from health survey).

Adverse reactions: The evaluation of adverse reactions refers to the Evaluation criteria of Common adverse events of the National Cancer Institute of the United States.^[13]^

### 2.8 Data collection and management

Nutrition intervention will run through the whole process of radiotherapy and chemotherapy. According to the outcome indicators, the data before radiotherapy and chemotherapy, every two weeks after the beginning of radiotherapy and chemotherapy, and after radiotherapy and chemotherapy are collected. All data will be collected by an assistant, and recorded in detail in the predesigned table. All the research data will be stored in a separate window, and only the researchers of this research group can access the relevant research data.

### 2.9 Statistical analysis

The data will be statistically analyzed by SPSS 16.0. The counting data will be expressed by rate, $\chi^{2}$ test will be used, and the measurement data will be expressed by $\bar{x}\pm s$. A *t* test will be used for comparison between groups. The difference is statistically significant when *P* < .05.

## 3 Discussion

Nutritional support has become an important part of tumor treatment. Ideal nutritional support should be based on the accurate assessment of the nutritional status of patients. ONS is a kind of nutrition supplement for special medical purpose, which can increase the intake of energy and nutrients. It is economical, convenient and easy to be accepted by patients.^[14]^ ONS is the preferred nutritional route recommended by both Chinese and European guidelines for tumor patients.^[15,16]^ Oral nutritional supplement plays an irreplaceable role in improving the tolerance of tumor patients, improving the nutritional status of tumor patients during radiotherapy, and even prolonging their life cycle.

Radiation esophagitis, dysphagia, and pain often occur in patients with esophageal cancer after radiotherapy, and malnutrition affects the course of treatment.^[17]^ Studies have shown that malnutrition is an independent risk factor for poor prognosis in patients with esophageal cancer.^[18]^ During radiotherapy and chemotherapy, many patients with esophageal cancer have difficulties in eating solid food, but an ordinary half-stream diet and liquid diet cannot meet their target energy requirements.^[3]^ Due to the hindrance of protein synthesis in the liver and abnormal protein metabolism, tumor patients are prone to decrease in varying degrees of protein (including albumin, prealbumin, transferrin).^[19]^

Although studies have confirmed that ONS can supplement the lack of energy and protein in patients with esophageal cancer, maintain and increase the weight of patients, improve nutritional status, and improve the quality of life,^[9]^ there is still a lack of strict RCTs to support this conclusion. In this study, a strict randomized controlled method will be used to evaluate the effect of oral nutritional supplement therapy on nutritional status and quality of life in patients with esophageal cancer undergoing radiotherapy and chemotherapy.

This study also has the following limitations: due to the factors of intervention, researchers and patients cannot strictly achieve double-blind, there may be a certain bias; there is a regional population included in this study, which may have a certain impact on the results.

## Author contributions

**Data collection:** Xiancui Wu and Xiaowei Wei.

**Funding support:** Xiuqin Ren.

**Investigation:** Xiaowei Wei.

**Resources:** Xiaowei Wei and Li Xu.

**Software operating:** Xiancui Wu and Xiuqin Ren.

**Supervision:** Yunxia Chen and Li Xu.

**Writing – original draft:** Yunxia Chen and Xiancui Wu.

**Writing – review & editing:** Yunxia Chen and Xiuqin Ren.
